# Physiological mechanisms of adaptive developmental plasticity in *Rana temporaria* island populations

**DOI:** 10.1186/s12862-017-1004-1

**Published:** 2017-07-07

**Authors:** Pablo Burraco, Ana Elisa Valdés, Frank Johansson, Ivan Gomez-Mestre

**Affiliations:** 1 Ecology, Evolution, and Development Group, Department of Wetland Ecology, Doñana Biological Station, CSIC, E-41092 Seville, Spain; 2 Department of Organismal Biology, Physiological Botany, Uppsala University, SE-75651 Uppsala, Sweden; 3 Department of Ecology and Genetics, Uppsala University, SE-75236 Uppsala, Sweden; 4 Department of Ecology, Environment and Plant Sciences, Stockholm University, SE-10691 Stockholm, Sweden; 5 Department of Ecology, Environment and Plant Sciences, Stockholm University, SE-10691 Stockholm, Sweden

**Keywords:** Amphibians, Corticosterone, Developmental plasticity, Evolutionary physiology, Oxidative stress, Telomere length

## Abstract

**Background:**

Adaptive plasticity is essential for many species to cope with environmental heterogeneity. In particular, developmental plasticity allows organisms with complex life cycles to adaptively adjust the timing of ontogenetic switch points. Size at and time to metamorphosis are reliable fitness indicators in organisms with complex cycles. The physiological machinery of developmental plasticity commonly involves the activation of alternative neuroendocrine pathways, causing metabolic alterations. Nevertheless, we have still incomplete knowledge about how these mechanisms evolve under environments that select for differences in adaptive plasticity. In this study, we investigate the physiological mechanisms underlying divergent degrees of developmental plasticity across *Rana temporaria* island populations inhabiting different types of pools in northern Sweden.

**Methods:**

In a laboratory experiment we estimated developmental plasticity of amphibian larvae from six populations coming from three different island habitats: islands with only permanent pools, islands with only ephemeral pools, and islands with a mixture of both types of pools. We exposed larvae of each population to either constant water level or simulated pool drying, and estimated their physiological responses in terms of corticosterone levels, oxidative stress, and telomere length.

**Results:**

We found that populations from islands with only temporary pools had a higher degree of developmental plasticity than those from the other two types of habitats. All populations increased their corticosterone levels to a similar extent when subjected to simulated pool drying, and therefore variation in secretion of this hormone does not explain the observed differences among populations. However, tadpoles from islands with temporary pools showed lower constitutive activities of catalase and glutathione reductase, and also showed overall shorter telomeres.

**Conclusions:**

The observed differences are indicative of physiological costs of increased developmental plasticity, suggesting that the potential for plasticity is constrained by its costs. Thus, high levels of responsiveness in the developmental rate of tadpoles have evolved in islands with pools at high but variable risk of desiccation. Moreover, the physiological alterations observed may have important consequences for both short-term odds of survival and long term effects on lifespan.

**Electronic supplementary material:**

The online version of this article (doi:10.1186/s12862-017-1004-1) contains supplementary material, which is available to authorized users.

## Background

Developmental plasticity occurs when an environmental input induces a lasting alteration in an organism’s phenotype. Developmental plasticity is generally a non-reversible process and can modulate acclimation capacity (reversible plasticity) in later developmental-stages [1]. Evolutionary benefits of adaptive developmental plasticity have been broadly assessed by both theoretical studies [2–4] and empirical approaches [5, 6], reviewed in [7]. Theoretical studies argue that developmental plasticity can increase population viability and facilitate the maintenance of genetic variation in two main ways: reducing the effect of genetic drift by moderating bottlenecks and shielding genetic variants from selection [8, 9]. This shielding effect may slow down the response to selection [10], but at the same time increases the odds of population persistence under environmental heterogeneity and preserves genetic variation hence granting a greater adaptive potential [9, 10]. Because plasticity confers a higher capacity for surviving new or rapidly changing environments, it can facilitate divergence among populations [11–13] and ultimately foster speciation [3, 8], as both empirical and theoretical studies have shown.

Environmental heterogeneity affects the degree of adaptive developmental plasticity, as predicted by theory [9, 14] and demonstrated empirically [15, 16]. Thus, heterogeneous environments are expected to select for highly plastic genotypes, whereas homogeneous environments would tend to reduce plasticity, especially if there are maintenance costs associated to such plasticity [17–19]. Populations diverging in the extent of their adaptive plastic responses as a consequence of selection under different environmental regimes would require qualitative or quantitative divergence in the mechanisms underlying phenotypic expression. The expression of alternative phenotypes relies on changes in gene expression [19, 20] and/or changes in the regulation of physiological pathways [21]. Thus, understanding the physiological mechanisms regulating developmental plasticity is key to understand the origin of evolved differences among populations in contrasting environments [22–24].

Developmental plasticity is particularly critical for amphibians because they are typically species with low vagility and high philopatry to highly variable habitats [25]. The majority of amphibians exhibit ancestral aquatic reproduction [26] and breed in temporary water bodies where the larvae develop until metamorphosis. A major larval-stage risk is pool drying and amphibian larvae can often detect fluctuations in water level and accelerate development in response to decreased water levels to achieve an early metamorphosis [27, 28]. Theory predicts that populations exposed to more fluctuating hydroperiods would exhibit greater developmental plasticity since it would allow tadpoles to adjust the timing of metamorphosis accelerating development when ponds dry up or extending the larval period if ponds persist for longer. However, the evolution of developmental plasticity might be constrained by costs of maintaining and/or producing such responses and also limited by factors such as reduced genetic variation or the impossibility of developing appropriate phenotypic responses at all points throughout development [29–31]. The populations of *Rana temporaria* on the Swedish islands are a paradigmatic example of the relationship between environmental heterogeneity and degree of adaptive plasticity [17]. These populations were established between 70 and 800 years ago [32] by frogs migrating from the mainland [33]. The islands have rocky pools where the frogs breed. These pools vary greatly and consistently in depth and size among islands, and consequently vary in average pond duration, ranging from permanent to ephemeral [17]. Over the last decade, several studies have analyzed the adaptive divergence in developmental rate among these *R. temporaria* populations. These studies have found signs of developmental canalization so that populations occupying islands with only ephemeral pools show overall faster developmental rates than populations from islands with permanent pools [34] while also showing reduced within-population genetic variation for developmental rate [34]. Moreover, island populations show marked differences in plasticity of their developmental rate [35] according to pond duration during the breeding season. In addition, [36] found evidence of costs of developmental plasticity, although these were only noticeable for the most plastic populations [36]. Faster developing populations were also found to express higher levels of thyroid hormone receptors (alpha and beta) and genes associated with higher metabolic activity [20]. This is congruent with mechanisms of developmental acceleration found in other species, namely increased thyroid hormone and corticosterone levels [37].

Developmental acceleration in amphibians is mediated by neuroendocrine pathways, especially by the hypothalamic pituitary adrenal axis (HPA) [38]. The HPA-axis is activated by external environmental inputs such as decreased water height [39], ultimately resulting in increased corticosterone production, which together with thyroid hormone synergistically activate or repress nuclear target genes causing increased metabolic rate, and accelerated morphogenesis [38]. Prolonged corticosterone secretion enhances metabolism and development [40, 41] but it can be at the cost of overproduction of toxic substances called reactive oxygen species (ROS) [42] that can inflict considerable cellular damage. Similarly, [43] found that bird species with faster development and smaller body size produced higher antioxidant enzymes. In our study, we expect that *R. temporaria* populations exposed to divergent regimes of pool drying will show adaptive differences in developmental rate. These among-population differences in developmental plasticity would be explained by altered gene expression associated with adaptive changes in metabolic activity and rate of morphogenesis, which ultimately would involve divergent corticosterone and antioxidant levels.

High ROS production also results in DNA damage, of which telomere shortening is of great importance because of its association with life-history trade-offs and lifespan [44, 45]. Telomeres are non-coding tandem repeat sequences of the terminal regions of the chromosomes with high G-C strand asymmetry [46]. Telomeres are determinants of cell senescence and are also involved in chromosome stability by avoiding chromosome fusion [47]. Telomere replication occurs via reverse transcriptase telomerase that adds telomeric repeats to 3′ overhang. However, telomere ends shorten over time after each cell division until critical telomere length is reached and initiates apoptosis, or programmed cell death [48]. Enhanced telomere abrasion has been described as a consequence of compensatory growth and developmental acceleration [49], mainly as a direct consequence of oxidative damage. In the wild, telomere shortening studies are principally focused on ageing [50, 51] and body condition quantification [52, 53]. However, only a few evolutionary studies include telomeres as a mechanism under selection even though telomere shortening correlates with numerous variations in life-history traits [54], and most of these studies have been conducted on humans [55–57].

Here we examine whether adaptive differences in developmental rate among *R. temporaria* populations are associated with changes in corticosterone levels, oxidative stress, and telomere length. We expected highly plastic populations to increase their corticosterone levels to a greater extent in response to pond drying than less plastic populations. We also hypothesized that populations with high developmental plasticity may pay a cost of maintaining such plastic ability in terms of higher constitutive levels of corticosterone and oxidative stress, and likely shorter telomeres, compared with less plastic populations.

## Methods

### Study area and field sampling

We studied the physiological consequences of adaptive divergence in developmental rate of *Rana temporaria* tadpoles from six islands located in the Gulf of Bothnia (Umea, Sweden) in a 10 km section of the coastline. The size range of the islands is between 9 and 38 ha. Frogs breed in water filled pools created by rocky depression on these islands. The pools on the islands differ in their water permanence such that some have ephemeral pools, other permanent pools, and others have a mixture of permanent and ephemeral pools. There is no relationship between predator abundance and life history traits among pools on these islands [32], and consequently pond duration seems to be the main environmental factor determining larval development in this system.

On 5 May 2014 we collected between 2 and 4 clutches from each of six islands. We separated the islands into three type of habitats according to their pool characteristics: two had only permanent pools (Storhaddingen 63° 40′N, 20° 25′ E, four clutches; Lillhaddingen 63° 40′N, 20° 24′E, two clutches), two only ephemeral pools (Sävar-Tärnögen 63° 45′N, 20° 36′E, four clutches; Ålgrundet 63° 41′N, 20° 25′E, two clutches) and two a mixture of permanent and ephemeral pools (Petlandsskär 63° 39′N, 20° 24′E, three clutches; Bredskär 63° 39′N, 20° 18′E, three clutches). Clutches were raised until Gosner stage 25 [58] separately in 1 L plastic containers (9.5 cm × 9.5 cm, height 10 cm) filled with 500 mL of reconstituted soft water (see [59] for details) in a climatic chamber at 14 °C and a light: dark cycle of 18 h: 6 h simulating natural photoperiod conditions.

### Experimental setup

One week after egg sampling tadpoles reached the free-feeding stage and started to swim actively (Gosner stage 25). At that time, 12 tadpoles per clutch (six tadpoles per treatment) for a total of 216 experimental units were individually transferred to 1 L containers filled with 750 mL of reconstituted soft water, where each tadpole was haphazardly assigned to an experimental treatment, i.e. constant water level or simulated pool drying. We renewed water every 4 days and tadpoles were fed ad libitum with lightly boiled spinach on each day of water change [60]. Containers were placed on shelves in a random order with respect to treatment and island in a walk-in climate chamber. Temperature and light were set to 22 °C and to 18 h: 6 h of light: dark. The experiment included a water level factor with two levels: constant water and simulating pool drying conditions. In the simulating pool drying treatment we decreased the initial water volume of 750 mL (10 cm) by 33% at each water change starting on day 5 until day 25, keeping the water volume constant at 66 mL (1 cm depth) afterwards. Water temperature did not differ significantly between treatments despite the differences in water level. The experiment ended when tadpoles reached Gosner stage 42 (front legs visible). We monitored tadpoles twice a day (09.00 and 21.00) to check for metamorphs. At this stage tadpoles were photographed to determine their length using ImageJ (version 1.47 t), and were weighed on a high precision balance to the nearest 0.1 mg. Clutch size from each island population varied between 2 and 4 (see Additional file 1).

### Tissue collection

Tadpoles at 42 Gosner were euthanized by immersion in a buffered solution of MS-222. A portion of muscle from the tadpoles’ tail was removed with a surgical blade and preserved at −20 °C for telomere analyses. The rest of the tail was snap frozen in liquid nitrogen and preserved at −80 °C until corticosterone assays were conducted. The rest of the body was also snap frozen for oxidative stress assays.

### Corticosterone assay

Corticosterone content was determined in tails (50–60 mg) collected from tadpoles at 42 Gosner Stage. Tissue was homogenized with an Ultraturrax TP18/10 (Hanke & Kunkel; IKA-Werk) during 30 s and the hormone was extracted following an organic phase extraction with 1 mL ethyl acetate during 30 min at 4 °C and continuous shaking. Samples were then centrifuged at 5000 rpm during 15 min and a known volume of the supernatant was taken and evaporated in a speedVac. Dried elutes were re-suspended in a final volume of 120 μL of the assay buffer provided with the enzymoinmunoassay kit supplemented with ethanol (<5% of final volume) to aid the re-suspension of steroids. We assayed each sample in duplicate (50 μL per sample) for corticosterone determination through specific enzymoinmunoassays (Arbor Assays, K014-H1/H5). The corticosterone antibody has low cross-reactivities to cortisol (0.38%), 11-desoxycorticosterone (12.3%), or progesterone (0.24%). The efficiency of the extraction was checked by spiking several aliquoted samples with 100 pg of exogenous corticosterone prior to extraction and comparing them to the non-spiked aliquotes. Recovery of exogenous corticosterone was never lower than 96%. The lowest point in the corticosterone standard curve was 2.29 pg/mL. To test for assay precision and variability, we determined the coefficient of variation (CV%) for intra- and inter-assay variation. Intra-assay variation was 8.74%. Inter-assay variation was 13.23% in the highest point of the standard curve and and 6.86% in the lowest point (*n* = 8 assays in both cases). The intra-sample correlation coefficient was 0.97. Corticosterone concentration was calculated from the %B/B0 curve by using the 4PLC fitting routine and following the online tool from [61].

### Oxidative stress assay

We quantified the activity of three antioxidant enzymes (catalase, glutathione peroxidase, and glutathione reductase). We also quantified malondialdehyde (MDA) concentration, a product formed during lipid peroxidation, total glutathione (GSH_t_) and the ratio of oxidized to reduced glutathione (GSH/GSSG ratio). After evisceration, tadpoles were individually homogenized with a Miccra homogenizer (Miccra D-1) at 35,000 rpm in a buffered solution to inhibit proteolysis (1:4; w:v; [62]). The homogenates were centrifuged at 14,000 rpm for 30 min at 4 °C and the resulting supernatants were aliquoted into six 0.6 mL tubes and cryopreserved at −80 °C. We determined total protein content by standard Bradford’s procedure [63]. The coefficient of variation in total protein determinations between duplicated samples was on average 4.27%.

Catalase (CAT) catalytic activity was indirectly quantified following [64]. According to this protocol, potassium permanganate (KMnO_4_) was used as an oxidizing agent that reacts with the catalase substrate hydrogen peroxide (H_2_O_2_) giving a red color that can be read at 480 nm 5 min after KMnO_4_ was added. We used commercial catalase (SIGMA – 60,634) for standard curves preparation. The coefficient of variation between duplicated samples was on average 3.26%. The intra-sample correlation coefficient was 0.95. Glutathione peroxidase (GPX) activity was quantified following the protocol developed by [65]. An excess of glutathione reductase (GR) reduces continually with NADPH the oxidized glutathione (GSSG) producing a constant level of reduced glutathione (GSH). We estimated NADPH oxidation spectrophotometrically at a wavelength of 340 nm. The coefficient of variation between duplicated samples was on average 2.12%. The intra-sample correlation coefficient was 0.88. GR activity was determined assessing the decrease in absorbance at 340 nm due to NADPH oxidation [66], and assays showed an average coefficient of variation between duplicated samples of 2.74%. The intra-sample correlation coefficient was 0.96. Lipid peroxidation was determined according to [67] by measuring MDA concentration. MDA is one product of the lipid peroxidation that reacts with thiobarbituric acids, generating a red product that absorbs at 535 nm. We quantified MDA concentration in nmol / mL by measuring optical density of each sample and then subtracting the blank values and comparing with the calibration curve. The coefficient of variation between duplicated samples was on average 3.93%. The intra-sample correlation coefficient was 0.95. Total glutathione (GSH_t_) was determined following [68]. Homogenates were diluted (1:10, w:v) and homogenized in a stock buffer solution (0.01 M PBS and 0.02 M EDTA). Three working solutions were prepared from the same stock buffer as follows: (a) 0.03 mM of NADPH, (b) 6 mM 5,5′-Dithiobis(2-nitrobenzoic acid) (DNTB), and (c) 50 units of GR/mL. Solution a and b were mixed using a ratio of 7:1 (A:B) and then 160 μL of this mixture was added to 40 μL of supernatant. After 15 s, 20 μL of the solution C were added and absorbance was read at 405 nm after 30 and 60 s. Total concentration of glutathione was determined by comparing changes in absorbance between the two readings, according to a standard curve generated by serial dilutions of glutathione from 1 nM to 0.031 nM. The repeatability was 90.3% (*n* = 10 samples).

### Relative telomere length quantification

Genomic DNA for telomere measurements was isolated using a high-salt DNA extraction protocol on a portion of tail muscle. All determinations were made from muscle tissue to avoid confounding differences between tissues in telomere length [69, 70]. Relative telomere length assays were performed using quantitative PCR (qPCR) following the protocol developed by [70] and calculated as the ratio of telomere repeats to a single-copy gene (T/S ratio; [71]). As a single copy gene we used glyceraldehyde-3-phosphate dehydrogenase (GAPDH, GenBank accession no. AF255390). Forward and reverse sequences of primers used to amplify GAPDH were 5-AACCAGCCAAGTACGATGACAT-3′ (GAPDH-F) and 5′-CCATCAGCAGCAGCCTTCA-3′ (GAPDH-R), respectively. Forward and reverse primers of the target gene were 5′CGGTTTGTTTGGGTTTGGGTTTGGGTTTGGGTTTGGGTT-3′ (Tel1b) and 5′-GGCTTGCCTTACCCTTACCCTTACCCTTACCCTTACCCT-3′ (Tel2b), respectively. We performed qPCR in two separate plates for GAPDH and telomere genes by adding 20 ng of genomic DNA from each sample. The set of primers used (Tel1b/Tel2b and GAPDH-F/GAPDH-R) was at an initial concentration of 900 nM containing 10 μL of Brilliant SYBR Green QPCR Master Mix (Roche) in a final volume of 20 μL. PCR protocol consisted of 10 min at 95 °C followed by 30 cycles of 1 min at 56 °C and 1 min at 95 °C for telomere fragment amplification, and 10 min at 95 °C followed by 40 cycles of 1 min at 60 °C and 1 min at 95 °C for GADPH fragment. We conducted qPCRs on a LightCycler 480 (Roche) and we tested the efficiency of each plate by performing a control standard curve by serially diluting a pool of samples from the different treatments and islands in triplicate (160, 40, 10, 2.5 and 0.66 ng of DNA per well). We calculated the cycle threshold (C_t_) value of each sample for each plate. Threshold is the basal level of fluorescence. C_t_ is defined as the number of cycles needed to detect a signal above the threshold. All samples were run in duplicate and relative telomere length was calculated following the formula [72]:

$$ \mathrm{T}/\mathrm{S}\ \mathrm{ratio}=\left[{{{\left({\mathrm{E}}_{\mathrm{t}\mathrm{elomere}}\right)}^{\Delta \mathrm{C}}}_{\mathrm{t}}}^{\mathrm{t}\mathrm{elomere}\ \left(\mathrm{control}\hbox{--} \mathrm{sample}\right)}\right]/\left[{{{\left({\mathrm{E}}_{\mathrm{GAPDH}}\right)}^{\Delta \mathrm{C}}}_{\mathrm{t}}}^{\mathrm{GAPDH}\ \left(\mathrm{control}\hbox{--} \mathrm{sample}\right)}\right] $$

where E_telomere_ is the qPCR efficiency of telomere fragment; E_GAPDH_ is the qPCR efficiency of the GAPDH fragment; ΔC_t_ telomere is the C_t_ deviation of control – sample of the telomere (target gene) fragment; ΔC_t_ GAPDH is the C_t_ deviation of control – sample of reference of GADPH (gene of reference) fragment. The coefficient of variation for duplicated samples was 0.65% on average for GAPDH assays and 1.36% for telomere assays. The intra-sample correlation coefficient was 0.90 and 0.86 for the telomere and GAPDH assays, respectively.

### Statistical analyses

Statistical analyses were conducted in R, version 3.3.1 (R Development Core Team). We checked whether residuals followed normal distributions conducting Kolgomorov-Smirnov tests (“lillie.test” in “nortest” package, version 1.0–2). We also tested for homoscedasticity using Barlett’s tests with the function “bartlett.test” (“car” package; version 2.0–22). We fitted linear and generalized mixed effect models using “lmer” function for normal data and “glmer” for non-normal data (package “lme4”; version 1.1–7). We used *water level* (constant or simulated pool drying) and *island habitat* as fixed factors, and included *clutch* nested within *island* as random factors in the models. We used likelihood ratio tests to determine the significance of each predictor. Days to metamorphosis, corticosterone, GPX, GR, GSH, and telomere data were log-transformed to meet parametric assumptions. We estimated growth rate as log (body mass at 42 Gosner stage) – log (days to reach 42 Gosner stage). Intraclass correlation coefficients were calculated using “ICCest” function (“ICC” package; version 2.3.0).

## Results

Survival was very high throughout the experiment (93.05%) and we found no significant differences between treatments, clutches, or island habitats.

**Fig. 1:**
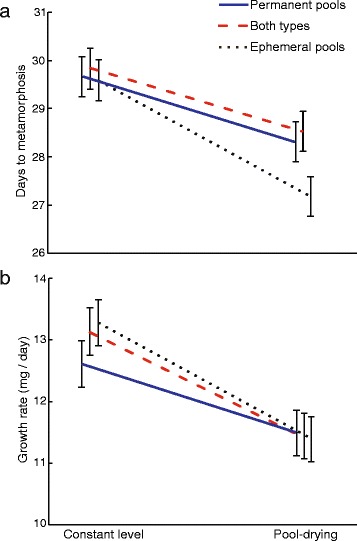
The effect of pool drying on (**a**) larval period and (**b**) growth rate in *Rana temporaria* tadpoles from three island habitats: islands with only permanent pools (*blue line*), islands with a mixture of ephemeral and permanent pools (*dashed red* line), and islands with only temporary pools (*dotted black line*). Data are least square means ± standard error

Simulated pool-drying induced accelerated development in *R. temporaria* larvae (χ^2^ = 58.05, *P* < 0.001; Fig. 1a), but the degree of response differed among island habitats as indicated by a significant island habitat by water level interaction (χ^2^ = 6.73, *P* = 0.035; Fig. 1a). Tadpoles from islands with only ephemeral pools showed the highest developmental plasticity since they reduced the time to metamorphosis by 8.13% on average, compared to tadpoles from islands with both pool-drying regimes and with only permanent pools, which accelerated their development by 4.40% and by 4.57% on average, respectively. Pool drying decreased larval growth rate by an average of 12.03% (χ^2^ = 42.39, *P* < 0.001; Fig. 1b). However, we did not find differences in growth rate among island habitats (χ^2^ = 0.49, *P* = 0.784) or in the interaction between island habitat and water level (χ^2^ = 1.99, *P* = 0.390; Fig. 1b). Also, pool drying decreased snout-to-vent length of the emerging metamorphs (χ^2^ = 51.01, *P* < 0.001), but we did not find differences in length among island types (χ^2^ = 0.31, *P* = 0.856) or in the interaction between island type and water level (χ^2^ = 2.15, *P* = 0.341).

Simulated pool desiccation induced endocrine responses in *R. temporaria* tadpoles, indicated by increased corticosterone levels by an average 25.48% (χ^2^ = 8.71, *P* = 0.003; Fig. S1 in Additional file 2). However, we did not find significant differences between island habitats or their interaction with water level (all *P* > 0.110; Fig. S1 in Additional file 2).

**Fig. 2:**
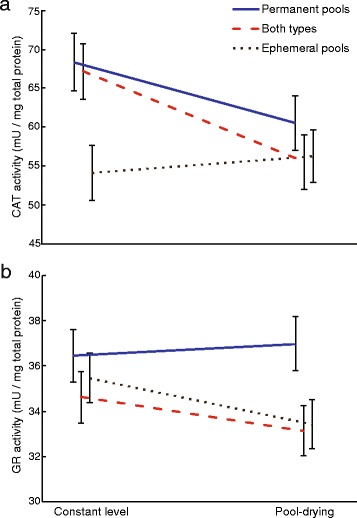
The effect of pool drying on (**a**) catalase (CAT) and (**b**) glutathione reductase (GR) activity on *Rana temporaria* tadpoles at 42 Gosner stage from three island habitats: islands with only permanent pools (*blue line*), islands with a mixture of ephemeral and permanent pools (*dashed red line*), and islands with only temporary pools (*dotted black line*). Data are least square means ± standard error

We also found alterations in antioxidant responses of the different populations in relation to water level. CAT activity decreased by 9.14% on average in tadpoles responding to pool drying (χ^2^ = 3.84, *P* = 0.049; Fig. 2a). Island habitat also affected CAT activity (χ^2^ = 6.56, *P* = 0.038; Fig. 2a) so that it was 11.21% and 16.86% lower in tadpoles from islands with ephemeral pools than in tadpoles from islands with both types of pools or with permanent pools, respectively. CAT levels were not affected by the interaction between pool drying and island habitat (χ^2^ = 4.37, *P* = 0.112; Fig. 2a). Pool drying seemed to induce a slight increase in GPX levels, but the effect was not significant (χ^2^ = 2.84 *P* = 0.092; Fig. S2 in Additional file 2). Neither island habitat nor its interaction with pool drying significantly affected GPX levels (χ^2^ = 3.25, *P* = 0.197, and χ^2^ = 0.01, *P* = 0.996, respectively; Fig. S2 in Additional file 2). Pool drying did not alter GR levels (χ^2^ = 2.371, *P* = 0.124; Fig. 2b). However, we found marginally non-significant differences between island habitats (χ^2^ = 5.78, *P* = 0.056; Fig. 2b). Tadpoles from islands with only ephemeral pools and from islands with both types of pools showed lower constitutive GR levels than tadpoles from permanent pools (6.26% and 7.84% lower GR levels on average, respectively). We did not find differences in the interaction between pool drying and island habitat in GR levels (χ^2^ = 1.18, *P* = 0.555; Fig. 2b).

We found that alterations in antioxidant enzymatic activity were not necessarily associated with cellular oxidative damage, since MDA values were not affected by pool drying, and showed no differences among island habitats, or their interaction (all *P* > 0.109; Fig. S3 in Additional file 2). Decreased water level resulted on average in 17.31% lower GSH_t_ values (χ^2^ = 9.49, *P* = 0.002). Levels of GSH_t_ was unaltered by either island habitat or the island habitat by water level interaction (χ^2^ = 1.90 *P* = 0.386 and χ^2^ = 4.69, *P* = 0.096, respectively; Fig. S4 in Additional file 2). The ratio of reduced to oxidized glutathione (GSH/GSSG ratio) was not affected by pool drying (χ^2^ = 3.16, *P* = 0.076; Fig. S5 in Additional file 2), and showed no differences among island habitats (χ^2^ = 2.54, *P* = 0.281; Fig. S5 in Additional file 2), or their interaction (χ^2^ = 0.36, *P* = 0.833; Fig. S5 in Additional file 2).

**Fig. 3:**
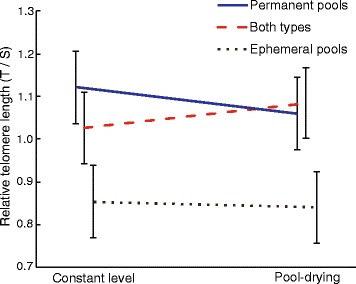
The effect of pool drying on relative telomere length (T/S ratio) of tail muscle tissue from *Rana temporaria* tadpoles at Gosner stage 42 from three island habitats: islands with only permanent pools (*blue line*), islands with a mixture of ephemeral and permanent pools (*dashed red line*), and islands with only temporary pools (*dotted black line*). Data are least square means ± standard error

Relative telomere length was not affected by water level treatment (χ^2^ = 0.01, *P* = 0.923; Fig. 3), but it varied significantly among island habitats (χ^2^ = 6.078, *P* = 0.048; Fig. 3). Tadpoles from ephemeral pools showed on average 22.31% shorter relative telomere length than tadpoles from permanent pools, and 19.89% shorter relative telomere length than tadpoles from semi-permanent pools. The interaction between relative telomere length and water level was not significant (χ^2^ = 1.46, *P* = 0.482; Fig. 3).

Relative telomere length and GSH_t,_ did not correlate with duration of larval period or growth rate (all *P* > 0.110). We found, however, a slight negative correlation between GSH/GSSG ratio and larval period (R^2^ = 0.089; *P* = 0.019).

## Discussion

Reaction norms can evolve under selection in divergent environmental regimes if plasticity confers adaptive value to individuals developing alternative phenotypes, but also if costs of plasticity select for less plastic genotypes in less variable environments [2, 3, 17]. Here we found evidence for divergence among *Rana temporaria* populations in their ability to accelerate development in response to decreased water level in accordance with the predominant pool drying regimes. The studied populations have been estimated to experience a small degree of neutral genetic differentiation [33], although *F*st estimates could have been biased downwards due to high heterozygosity of the markers used [73, 74] and hence population differentiation may be greater than previously thought. Populations from islands with only ephemeral pools showed the greatest capacity for developmental acceleration in response to pool drying. This indicates that selection on the regulation of developmental rate under different flooding regimes has resulted in increased plasticity in these populations (genetic accommodation), not in canalized fast development or genetic assimilation [2, 75]. We suspect that such a steep reaction norm is derived within the species, but phylogenetic reconstructions of the ancestral state of plasticity for these populations would be required to resolve the polarity of the changes in our *Rana temporaria* populations. Selection for rapid larval development can result in canalized fast development and loss of plasticity [20, 76]. In our study, populations inhabiting islands with ephemeral pools have evolved greater developmental plasticity. Our results differ somewhat from a previous study on this system [17], where islands with both types of pools showed higher plasticity. This discrepancy can be a consequence of broader than expected interannual variability in pool duration [17], among-population variation in costs of plasticity maintenance [30], and/or stochastic effects of sampling different genotypes over different years.

The mechanisms underlying developmental acceleration in amphibians are well known, and rely on activation of the hypothalamic-pituitary-axis [27, 38]. This neuroendocrine activation in amphibians results in higher thyroid hormone and corticosterone levels, which enhance cell replication rate [54] and morphogenesis [38]. We observed that larvae from all populations increased corticosterone levels to a similar extent (around 25%) when facing decreased water levels. Such up-regulation of corticosterone explains the ability of these populations to accelerate development, but does not reflect the observed among-population differences in their degree of plasticity. In contrast, the activity of antioxidant enzymes varied significantly among populations, which would suggest that populations under divergent environments evolved metabolic differences resulting in different levels of ROS production. In this case, highly plastic *Rana* populations showed lower constitutive levels of both CAT and GR enzymes than less plastic populations. CAT transforms hydrogen peroxide to water and oxygen whereas GR reduces glutathione disulfide to the sulfhydryl form of glutathione, both processes being essential in protecting cells from oxidative damage. Low enzymatic levels are associated with low production of ROS, but also with exhaustion of the enzymes as a result of oxidative stress via toxic substances or ROS production [77–79]. In addition, [20] found an increase in the gene transcript of catalase in tadpoles facing pool drying. Levels of catalase transcript were higher in populations that developed faster, supporting the idea of exhausted levels of these enzymes in populations permanently exposed to pool drying conditions. Thus, lower activities of CAT and GR might indicate an enzymatic inactivation caused by an excess of ROS produced during developmental acceleration [45, 80]. However, the absence of among-population differences in MDA or GSH levels do not fully support these conclusions since it indicates a lack of intense lipid peroxidation or antioxidant responses. Therefore, an alternative explanation might be that selection favored individuals that maximized ROS production when they experienced higher metabolic rates, hence showing lower antioxidant activity [81, 82].

Telomere length varied among populations adapted to different pool-drying regimes by evolving different extents of developmental plasticity. This is a novel and intriguing finding and it may help to understand the implications of developmental plasticity to lifespan and fitness. Telomere shortening occurs after each cell replication so that telomeres usually shorten with age [83, 84]. However, telomere shortening is also linked to increased metabolism [54], particularly to oxidative stress originated during intense cell respiration [54, 85]. In wild populations, telomere shortening has also been described as a predictor of mortality [86], reproductive costs [87] or the impact of infections [88]. Therefore, telomere shortening can result a reliable indicator of the biological age of individuals [86]. In our experiment, antioxidant responses observed in *Rana* populations from islands with only ephemeral pools suggest a high ROS production derived from intense metabolism, which is associated with the attrition of telomeres. Thus, based on our oxidative stress and telomere length determinations, we would expect accelerated development to bear the consequence of reduced lifespan and hence possibly reduced fitness. Among-population differences in telomere length have been associated to trans-generational effects of male age at reproduction due to a progressive elongation of telomeres in sperm with age [89–91]. Analogous maternal age effects have not been found [90]. Parental exposure to stressful conditions is also relevant to inheritance of telomere length, although these processes remain poorly understood [92]. In our study, shorter telomeres in tadpoles from islands with only ephemeral pools might be related to early age of first reproduction of males, which could be a long-term consequence of accelerated development against pool drying. However, further empirical studies will elucidate underlying mechanisms in telomere inheritance. Evaluations of telomerase activity and long-term studies testing the effects of parental telomere shortening on life-history traits of offspring will help to understand telomeric dynamic across generations.

Physiological differences among populations found in this study suggest that increased developmental plasticity may be associated with metabolic alterations that compromise the health and lifespan of individuals, as populations differed in antioxidant enzymes activity and length of terminal chromosomal regions. Reduced individual lifespan could have cascading demographic effects on population viability, although this remains to be explored.

## Conclusions

In sum, populations evolving in contrasting environments showed divergent levels of developmental plasticity and associated oxidative stress and telomere length variation, despite the slight neutral genetic differentiation previously described. These results emphasize the importance of including physiological measurements in the study of phenotypic plasticity, in order to be able to understand the underlying mechanisms of particular evolutionary and ecological processes.

## Additional files

Additional file 1: Raw data obtained during the study and used in statistical analyses. (XLSX 114 kb)

Additional file 2: Supplementary figures and corresponding legends. (PDF 501 kb)

## Abbreviations

CAT: Catalase
GPx: Glutathione peroxidase
GR: Glutathione reductase
GSH/GSSG ratio: Reduced/oxidized glutathione ratio
GSH_t_: Total glutathione
GSSG: Oxidized glutathione
MDA: Malondialdehyde
qPCR: Quantitative PCR

## References

[1] Beaman JE, White CR, Seebacher F (2016). Evolution of plasticity: mechanistic link between development and reversible acclimation. Trends Ecol Evol.

[2] West-Eberhard MJ (2003). Developmental plasticity and evolution.

[3] Pfennig DW, Wund MA, Snell-Rood EC, Cruickshank T, Schlichting CD, Moczek AP (2010). Phenotypic plasticity’s impacts on diversification and speciation. Trends Ecol Evol.

[4] Snell-Rood EC (2013). An overview of the evolutionary causes and consequences of behavioural plasticity. Anim Behav.

[5] Aubret F, Bonnet X, Shine R (2007). The role of adaptive plasticity in a major evolutionary transition: early aquatic experience affects locomotor performance of terrestrial snakes. Funct Ecol.

[6] Touchon JC, Warkentin KM. (2008). Reproductive mode plasticity: aquatic and terrestrial oviposition in a treefrog. Proc Natl Acad Sci U S A. 2008;105:7495-7499. 10.1073/pnas.0711579105 PMC2396680 18495921

[7] Moczek AP, Sultan S, Foster S, Ledón-Rettig C, Dworkin I, Nijhout HF, Abouheif E, Pfennig DW (2011). The role of developmental plasticity in evolutionary innovation. Proc R Soc Lond B.

[8] Draghi JA, Whitlock MC (2012). Phenotypic plasticity facilitates mutational variance, genetic variance, and evolvability along the major axis of environmental variation. Evolution.

[9] Gomez-Mestre I, Jovani R (2013). A heuristic model on the role of plasticity in adaptive evolution: plasticity increases adaptation, population viability and genetic variation. Proc R Soc Lond B.

[10] Price TD, Qvarnström A, Irwin DE (2003). The role of phenotypic plasticity in driving genetic evolution. Proc R Soc Lond B.

[11] Levin DA (2009). Flowering-time plasticity facilitates niche shifts in adjacent populations. New Phytol.

[12] Snell-Rood EC, Van Dyken JD, Cruickshank T, Wade MJ, Moczek AP (2010). Toward a population genetic framework of developmental evolution: the costs, limits, and consequences of phenotypic plasticity. BioEssays.

[13] Svanbäck R, Schluter D (2012). Niche specialization influences adaptive phenotypic plasticity in the threespine stickleback. Am Nat.

[14] Chevin LM, Lande R (2013). Evolution of discrete phenotypes from continuous norms of reaction. Am Nat.

[15] Gianoli E, González-Teuber M (2005). Environmental heterogeneity and population differentiation in plasticity to drought in *Convolvulus chilensis* (*Convolvulaceae*). Evol Ecol.

[16] Baythavong BS (2011). Linking the spatial scale of environmental variation and the evolution of phenotypic plasticity: selection favors adaptive plasticity in fine-grained environments. Am Nat.

[17] Lind MI, Johansson F (2007). The degree of adaptive phenotypic plasticity is correlated with the spatial environmental heterogeneity experienced by island populations of *Rana temporaria*. J Evol Biol.

[18] Beldade P, Mateus ARA, Keller RA (2011). Evolution and molecular mechanisms of adaptive developmental plasticity. Mol Ecol.

[19] Schlichting CD, Wund MA (2014). Phenotypic plasticity and epigenetic marking: an assessment of evidence for genetic accommodation. Evolution.

[20] Johansson F, Veldhoen N, Lind MI, Helbing CC (2013). Phenotypic plasticity in the hepatic transcriptome of the European common frog (*Rana temporaria*): the interplay between environmental induction and geographical lineage on developmental response. Mol Ecol.

[21] Ricklefs RE, Wikelski M (2002). The physiology/life-history nexus. Trends Ecol Evol.

[22] Valladares F, Balaguer L, Martinez-Ferri E, Perez-Corona E, Manrique E (2002). Plasticity, instability and canalization: is the phenotypic variation in seedlings of sclerophyll oaks consistent with the environmental unpredictability of Mediterranean ecosystems?. New Phytol.

[23] West-Eberhard MJ (2005). Developmental plasticity and the origin of species differences. Proc Natl Acad Sci U S A.

[24] Whitehead A, Roach JL, Zhang S, Galvez F (2011). Genomic mechanisms of evolved physiological plasticity in killifish distributed along an environmental salinity gradient. Proc Natl Acad Sci U S A.

[25] Smith MA, Green DM (2005). Dispersal and the metapopulation paradigm in amphibian ecology and conservation: are all amphibian populations metapopulations?. Ecography.

[26] Gomez-Mestre I, Pyron RA, Wiens JJ (2012). Phylogenetic analyses reveal unexpected patterns in the evolution of reproductive modes in frogs. Evolution.

[27] Denver RJ (1997). Proximate mechanisms of phenotypic plasticity in amphibian metamorphosis. Amer Zool.

[28] Richter-Boix A, Llorente GA, Montori A (2006). A comparative analysis of the adaptive developmental plasticity hypothesis in six Mediterranean anuran species along a pond permanency gradient. Evol Ecol Res.

[29] DeWitt TJ, Sih A, Wilson DS (1998). Costs and limits of phenotypic plasticity. Trends Ecol Evol.

[30] Auld JR, Agrawal AA, Relyea RA (2010). Re-evaluating the costs and limits of adaptive phenotypic plasticity. Proc R Soc Lond B.

[31] Murren CJ, Auld JR, Callahan H, Ghalambor CK, Handelsman CA, Heskel MA (2015). Constraints on the evolution of phenotypic plasticity: limits and costs of phenotype and plasticity. Heredity.

[32] Johansson F, Hjelm J, Giles BE (2005). Life history and morphology of *Rana temporaria* in response to pool permanence. Evol Ecol Res.

[33] Lind MI, Ingvarsson PK, Johansson H, Hall D, Johansson F (2011). Gene flow and selection on phenotypic plasticity in an island system of *Rana temporaria*. Evolution.

[34] Lind MI, Persbo F, Johansson F (2008). Pool desiccation and developmental thresholds in the common frog, *Rana temporaria*. Proc R Soc Lond B.

[35] Lind MI, Johansson F (2011). Testing the role of phenotypic plasticity for local adaptation: growth and development in time-constrained *Rana temporaria* populations. J Evol Biol.

[36] Lind MI, Johansson F (2009). Costs and limits of phenotypic plasticity in island populations of the common frog *Rana temporaria* under divergent selection pressures. Evolution.

[37] Gomez-Mestre I, Kulkarni S, Buchholz DR (2013). Mechanisms and consequences of developmental acceleration in tadpoles responding to pond drying. PLoS One.

[38] Denver RJ (2009). Stress hormones mediate environment-genotype interactions during amphibian development. Gen Comp Endocrinol.

[39] Boorse GC, Denver RJ (2003). Endocrine mechanisms underlying plasticity in metamorphic timing in spadefoot toads. Integr Comp Biol.

[40] Hayes TB, Wu TH (1995). Interdependence of corticosterone and thyroid hormones in toad larvae (*Bufo boreas*). II. Regulation of corticosterone and thyroid hormones. *J. Exp. Zool. A. Ecol.* Genet. Physiol.

[41] Wack CL, DuRant SE, Hopkins WA, Lovern MB, Feldhoff RC, Woodley SK (2012). Elevated plasma corticosterone increases metabolic rate in a terrestrial salamander. Comp Biochem Physiol A Mol Integr Physiol.

[42] Costantini D, Marasco V, Møller AP (2011). A meta-analysis of glucocorticoids as modulators of oxidative stress in vertebrates. J Comp Physiol B.

[43] Cohen AA, McGraw KJ, Wiersma P, Williams JB, Robinson WD, Robinson TR, Brawn JD, Ricklefs RE (2008). Interspecific associations between circulating antioxidant levels and life-history variation in birds. Amer Nat.

[44] Von Zglinicki T (2002). Oxidative stress shortens telomeres. Trends Biochem Sci.

[45] Monaghan P, Metcalfe NB, Torres R (2009). Oxidative stress as a mediator of life history trade-offs: mechanisms, measurements and interpretation. Ecol Lett.

[46] Blackburn EH (1991). Structure and function of telomeres. Nature.

[47] O’Sullivan RJ, Karlseder J (2010). Telomeres: protecting chromosomes against genome instability. Nat Rev Mol Cell Biol.

[48] Campisi J (2003). Cancer and ageing: rival demons?. Nat Rev Cancer.

[49] Metcalfe NB, Monaghan P (2011). Compensation for a bad start: grow now, pay later?. Trend Ecol Evol.

[50] Haussmann MF, Vleck CM (2002). Telomere length provides a new technique for aging animals. Oecologia.

[51] Bize P, Criscuolo F, Metcalfe NB, Nasir L, Monaghan P (2009). Telomere dynamics rather than age predict life expectancy in the wild. Proc R Soc Lond B.

[52] Trusina A (2014). Stress induced telomere shortening: longer life with less mutations?. BMC Syst Biol.

[53] Boonekamp JJ, Mulder GA, Salomons HM, Dijkstra C, Verhulst S. 2014. Nestling telomere shortening, but not telomere length, reflects developmental stress and predicts survival in wild birds. Proc R Soc Lond B. 2014;281:20133287. 10.1098/rspb.2013.3287 PMC4024283 24789893

[54] Haussmann MF, Marchetto NM (2010). Telomeres: linking stress and survival, ecology and evolution. Curr Zool.

[55] Seluanov A, Chen Z, Hine C, Sasahara TH, Ribeiro AA, Catania KC (2007). Telomerase activity coevolves with body mass not lifespan. Aging Cell.

[56] Gorbunova V, Seluanov A (2009). Coevolution of telomerase activity and body mass in mammals: from mice to beavers. Mech Ageing Dev.

[57] Eisenberg DT (2011). An evolutionary review of human telomere biology: the thrifty telomere hypothesis and notes on potential adaptive paternal effects. Am J Hum Biol.

[58] Gosner KL (1960). A simplified table for staging anuran embryos and larvae with notes on identification. Herpetologica.

[59] Räsänen KR, Laurila A, Merilä J (2003). Geographic variation in acid stress tolerance of the moor frog, *Rana arvalis*. I. Local adaptation. Evolution.

[60] Richter-Boix A, Orizaola G, Laurila A (2014). Transgenerational phenotypic plasticity links breeding phenology with offspring life-history. Ecology.

[61] http://www.myassays.com/arbor-assays-corticosterone-enzyme-immunoassay-kit.assay (Accessed 19 June 2017).

[62] Burraco P, Gomez-Mestre I (2016). Physiological stress responses in amphibian larvae to multiple stressors reveal marked anthropogenic effects even below lethal levels. Physiol Biochem Zool.

[63] Bradford MM (1976). A rapid and sensitive method for the quantitation of microgram quantities of protein utilizing the principle of protein-dye binding. Anal Biochem.

[64] Cohen G, Somerson NL (1969). Catalase-aminotriazole method for measuring secretion of hydrogen peroxide by microorganisms. J Bacteriol.

[65] Paglia DE, Valentine WN (1967). Studies on the quantitative and qualitative characterization of erythrocyte glutathione peroxidase. J Lab Clin Med.

[66] Cribb AE, Leeder JS, Spielberg SP (1989). Use of a microplate reader in an assay of glutathione reductase using 5, 5′-dithiobis (2-nitrobenzoic acid). Anal Biochem.

[67] Buege JA, Aust SD (1978). Microsomal lipid peroxidation. Method Enzymol.

[68] Galván I, Gangoso L, Grande JM, Negro JJ, Rodríguez A, Figuerola J, Alonso-Alvarez C (2010). Antioxidant machinery differs between melanic and light nestlings of two polymorphic raptors. PLoS One.

[69] Friedrich U, Griese EU, Schwab M, Fritz P, Thon KP, Klotz U (2000). Telomere length in different tissues of elderly patients. Mech Ageing Dev.

[70] Dlouha D, Maluskova J, Lesna IK, Lanska V, Hubacek JA (2014). Comparison of the relative telomere length measured in leukocytes and eleven different human tissues. Physiol Res.

[71] Cawthon RM (2002). Telomere measurement by quantitative PCR. Nucleic Acids Res.

[72] Pfaffl MW (2001). A new mathematical model for relative quantification in real-time RT–PCR. Nucleic Acids Res.

[73] Jost L (2008). GST and its relatives do not measure differentiation. Mol Ecol.

[74] Edelaar P, Burraco P, Gomez-Mestre I (2011). Comparisons between QST and FST—how wrong have we been?. Mol Ecol.

[75] Crispo E (2007). The Baldwin effect and genetic assimilation: revisiting two mechanisms of evolutionary change mediated by phenotypic plasticity. Evolution.

[76] Gomez-Mestre I, Buchholz DR (2006). Developmental plasticity mirrors differences among taxa in spadefoot toads linking plasticity and diversity. Proc Natl Acad Sci U S A.

[77] Barata C, Navarro JC, Varo I, Riva MC, Arun S, Porte C (2005). Changes in antioxidant enzyme activities, fatty acid composition and lipid peroxidation in *Daphnia magna* during the aging process. Comp Biochem Physiol B Biochem Mol Biol.

[78] Srinivasan R, Chandrasekar MJN, Nanjan MJ, Suresh B (2007). Antioxidant activity of *Caesalpinia digyna* root. J Ethnopharmacol.

[79] Slos S, Stoks R (2008). Predation risk induces stress proteins and reduces antioxidant defense. Funct Ecol.

[80] D’Autréaux B, Toledano MB (2007). ROS as signalling molecules: mechanisms that generate specificity in ROS homeostasis. Nat Rev Mol Cell Biol.

[81] Salin K, Auer SK, Rudolf AM, Anderson GJ, Cairns AG, Mullen W (2015). Individuals with higher metabolic rates have lower levels of reactive oxygen species in vivo. Biol Lett.

[82] Salin K, Auer SK, Rey B, Selman C, Metcalfe NB (2015). Variation in the link between oxygen consumption and ATP production, and its relevance for animal performance. Proc R Soc B.

[83] Frenck RW, Blackburn EH, Shannon KM (1998). The rate of telomere sequence loss in human leukocytes varies with age. Proc Natl Acad Sci U S A.

[84] Asghar M, Bensch S, Tarka M, Hansson B, Hasselquist D (2015). Maternal and genetic factors determine early life telomere length. Proc R Soc Lond B.

[85] Monaghan P, Charmantier A, Nussey DH, Ricklefs RE (2008). The evolutionary ecology of senescence. Funct Ecol.

[86] Barrett EL, Burke TA, Hammers M, Komdeur J, Richardson DS (2013). Telomere length and dynamics predict mortality in a wild longitudinal study. Mol Ecol.

[87] Bauch C, Becker PH, Verhulst S (2013). Telomere length reflects phenotypic quality and costs of reproduction in a long-lived seabird. Proc R Soc Lond B.

[88] Asghar M, Palinauskas V, Zaghdoudi-Allan N, Valkiūnas G, Mukhin A, Platonova E (2016). Parallel telomere shortening in multiple body tissues owing to malaria infection. Proc R Soc Lond B.

[89] Baird DM, Britt-Compton B, Rowson J, Amso NN, Gregory L, Kipling D (2006). Telomere instability in the male germline. Hum Mol Gen.

[90] Kimura M, Cherkas LF, Kato BS, Demissie S, Hjelmborg JB, Brimacombe M (2008). Offspring’s leukocyte telomere length, paternal age, and telomere elongation in sperm. PLoS Genet.

[91] Eisenberg DT, Hayes MG, Kuzawa CW (2012). Delayed paternal age of reproduction in humans is associated with longer telomeres across two generations of descendants. Proc Natl Acad Sci U S A.

[92] Haussmann MF, Heidinger BJ (2015). Telomere dynamics may link stress exposure and ageing across generations. Biol Lett.

