# Supplementary figures and images for: Physiological mechanisms of adaptive developmental plasticity in Rana temporaria island populations
Source: BMC Evol Biol. 2017 Jul 7;17:164. doi: 10.1186/s12862-017-1004-1 (PMC5501514; doi:10.1186/s12862-017-1004-1)

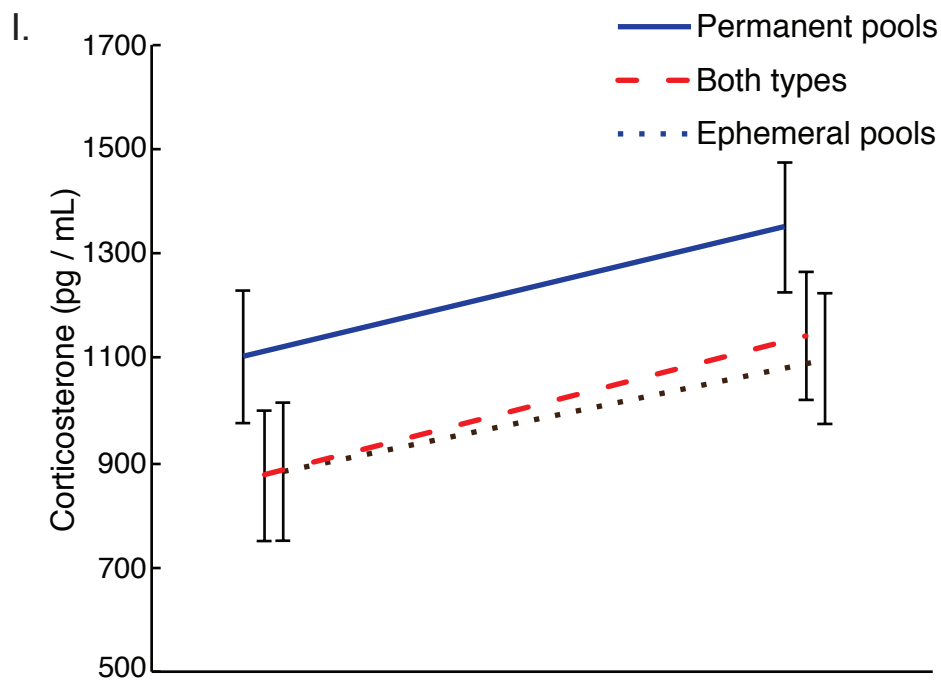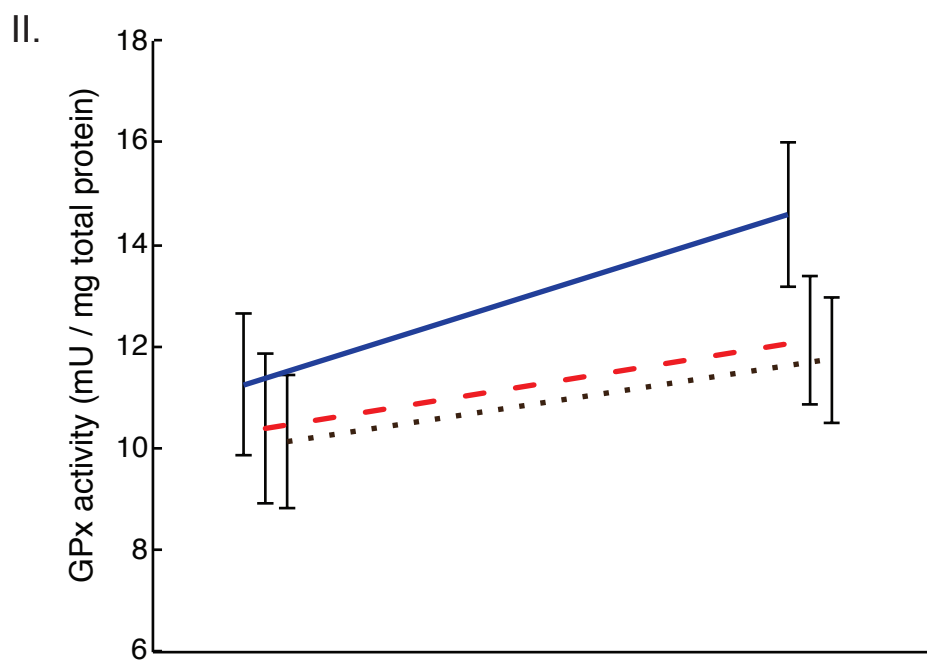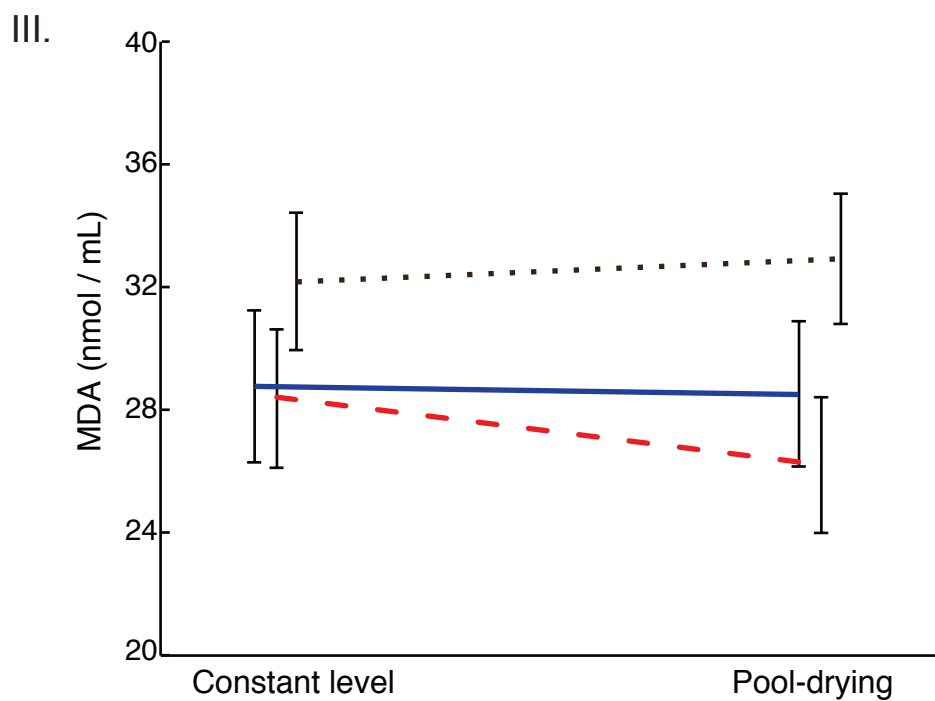

IV.

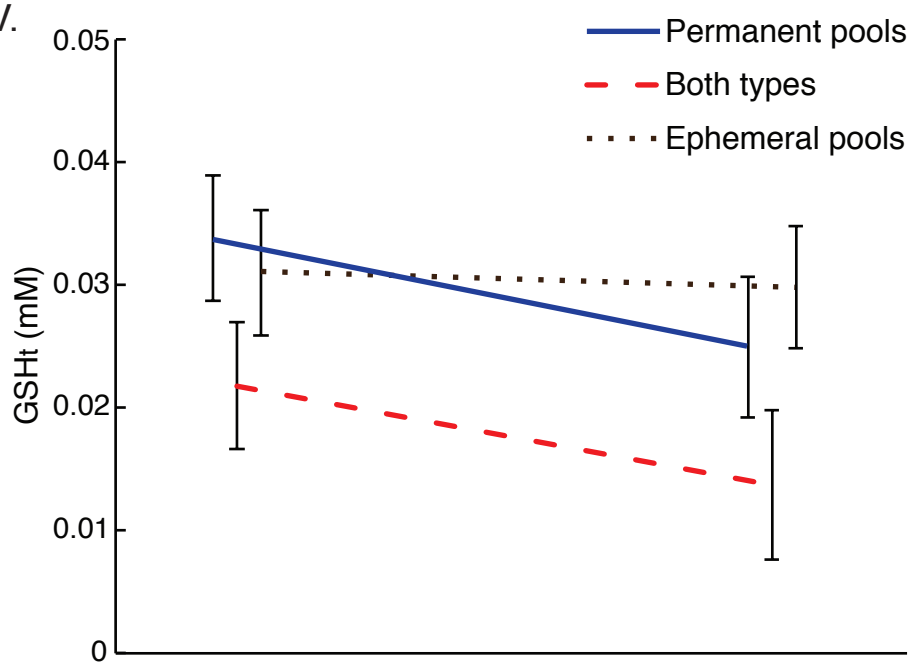

V.

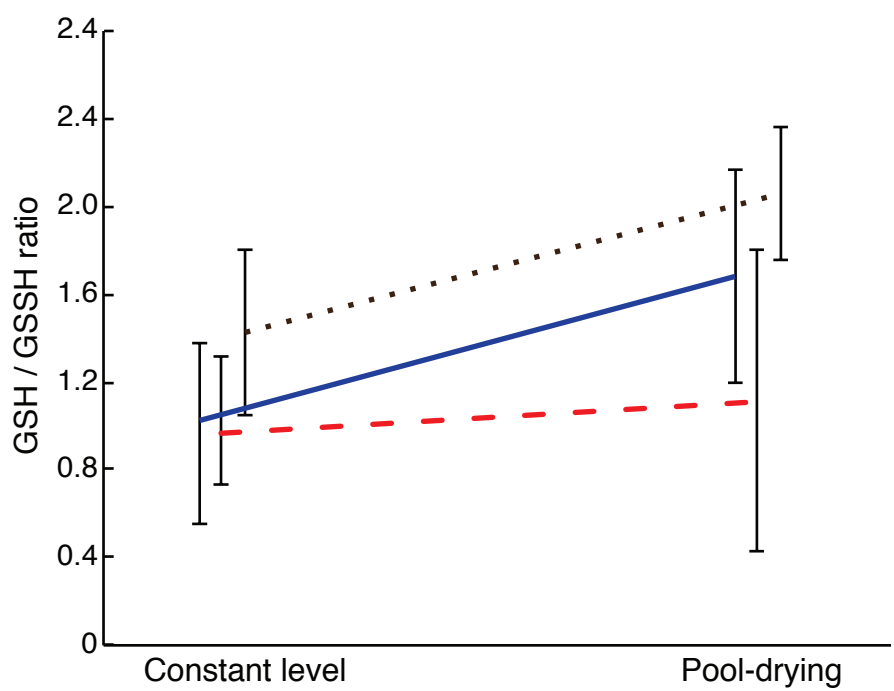

Supplement: Supplementary file 2 — Supplementary figures and corresponding legends. (PDF 501 kb) [file 12862_2017_1004_MOESM2_ESM.pdf]
